# Saving Fuel

**Published:** 1864-10

**Authors:** 


### SAVING FUEL.

On inquiry with a number of gentlemen, one ton of range coal is allowed for the cooking and washing purposes of the household for one month on an average during the year. This is double what it ought to be, and with coal costing at the time it is put into bins, including the necessary "kindling" of paper and wood, fifteen dollars a ton, amounts to ninety dollars a year. These statements are made as the result of experiments carried on under our own eye for the double pleasure of economizing and the greater one of knowing a thing personally for ourself and of being able to communicate the knowledge to others, to clergymen for example who are almost always too scantily paid and with whom one half the above amount is of very considerable importance. We obtained one of Fish's patent cooking lamps and have been using it for two seasons. A ton of coal was purchased on the 17th of June last; the next on the third of September, making two months and a half. Fire was regularly kindled and kept burning for three days in the week for cooking, washing, ironing and bread baking, to wit, on Mondays, Wednesdays and Saturdays; at other times the cooking for a family averaging ten persons was done with a gas lamp from Russell's establishment at 206 Pearl Street, New-York. The amount of gas used, for cooking purposes only, during that two months and a half, at $2.50 per thousand feet, was less than two dollars. The gas stove costs now $7.50, the largest size made being a No. four; it does all the cooking needed in a family, except baking bread, roasting meats and preparing pastries. Boiling water, boiling vegetables and frying meats can all go on at the same time. Bread can be toasted and buckwheat or batter or other thin cakes are easily made. It might be arranged for a great part of the winter that the range should be lighted for cooking every day's dinner, and use the gas or kerosene stove for breakfast, and thus, instead of having the range red hot from day light until bed time it really need not burn longer than three hours, for who ever knew a servant who could cook or wash or even make a cup of coffee for breakfast without a red hot stove. The difference between burning coal under such circumstances three hours instead of sixteen, is not a small item. N. B. Since writing the above, we chanced to call in at Mr. Russell's establishment, and have been exceedingly gratified to find that he has gotten up a family cooking stove, heated by kerosene or gas, at a cost of ten or twelve dollars each, which will roast ten pounds of beef, bake bread, pies, pastries, &c., with the greatest facility. In these days of costly fuel, this stove is a public godsend. Kerosene at one dollar a gallon will heat this stove for all necessary cooking purposes, at four cents an hour. The Flat Iron Heater is kept in operation for less than three cents an hour, and costs eight dollars; saving the suffocating heat of midsummer, which citizens have to endure on "ironing days."

